# Translation of patients’ advance directives in intensive care units: are we there yet?

**DOI:** 10.1186/s40560-023-00705-z

**Published:** 2023-11-15

**Authors:** Sira M. Baumann, Natalie J. Kruse, Paulina S. C. Kliem, Simon A. Amacher, Sabina Hunziker, Tolga D. Dittrich, Fabienne Renetseder, Pascale Grzonka, Raoul Sutter

**Affiliations:** 1 Intensive Care Unit, Department of Acute Medical Care, University Hospital Basel, Basel, Switzerland; 2 Department of Clinical Research, University of Basel, Basel, Switzerland; 3 Medical Communication and Psychosomatic Medicine, University Hospital Basel, Basel, Switzerland; 4 Medical Faculty, University of Basel, Basel, Switzerland; 5 Department of Neurology and Stroke Center, Cantonal Hospital St. Gallen, St. Gallen, Switzerland; 6 Department of Neurology and Stroke Center, University Hospital Basel and University of Basel, Basel, Switzerland

**Keywords:** Advance directives, Patients’ will, Intensive care, Neurocritical illness

## Abstract

**Objectives:**

This review examined studies regarding the implementation and translation of patients’ advance directives (AD) in intensive care units (ICUs), focusing on practical difficulties and obstacles.

**Methods:**

The digital PubMed and Medline databases were screened using predefined keywords to identify relevant prospective and retrospective studies published until 2022.

**Results:**

Seventeen studies from the United States, Europe, and South Africa (including 149,413 patients and 1210 healthcare professionals) were identified. The highest prevalence of ADs was described in a prospective study in North America (49%), followed by Central Europe (13%), Asia (4%), Australia and New Zealand (4%), Latin America (3%), and Northern and Southern Europe (2.6%). While four retrospective studies reported limited effects of ADs, four retrospective studies, one survey and one systematic review indicated significant effects on provision of intensive care, higher rates of do-not-resuscitate orders, and care withholding in patients with ADs. Four of these studies showed shorter ICU stays, and lower treatment costs in patients with ADs. One prospective and two retrospective studies reported issues with loss, delayed or no transmission of ADs. One survey revealed that 91% of healthcare workers did not regularly check for ADs. Two retrospective studies and two survey revealed that the implementation of directives is further challenged by issues with their applicability, phrasing, and compliance by the critical care team and family members.

**Conclusions:**

Although ADs may improve intensive- and end-of-life care, insufficient knowledge, lack of awareness, poor communication between healthcare providers and patients or surrogates, lack of standardization of directives, as well as ethical and legal concerns challenge their implementation.

## Background

Living wills were conceptualized in the 1960s by the American lawyer Luis Kutner after a student followed his cancer-stricken mother's directive and shot her in a hospital [1]. Today, patients’ advance directives are measures of self-determination, enabling individuals capable of judgement to determine their preferred medical treatment and interventions in case of severe illness and loss of judgment. This is of particular importance in the intensive care setting, where urgent treatment and end-of-life decisions can significantly impact patients’ outcomes. However, at this point of care, more than 95% of the critically ill are no longer capable of judgment and of making such pivotal decisions [2], a challenge that is particularly pronounced in the neurocritically ill patients [3]. Advance care planning is expected to gain more and more significance in the near future. Already in 1999, one in five deaths in the United States of America (USA) occurred after provision of intensive care [4]. This results in a projected ICU capacity expansion or improvement in advance care planning needed to accommodate for the expected doubling of the population over age 65 by 2030 [4]. In a recent study investigating health care utilization in dying cancer patients, 27.2% of cancer patients in the USA were admitted at least once to intensive care in their last 30 days of life, highlighting the importance of advance directives in this vulnerable patient population [5].

However, there has been limited focus on the implementation and translation of advance directives in intensive care worldwide. The lack of such attention can pose significant challenges for the ICU staff, particularly in situations like the SARS-CoV-2 pandemic where intensive care resources were depleted[6]. This problem might be further exacerbated by inadequate knowledge about advance directives challenging the effective allocation of resources. The expected rise in survivors with severe impairments that require prolonged rehabilitation further underscores the importance of an effective implementation of patient’s wishes and advance directives into every day critical care.

This review aims to examine studies and practices regarding the implementation and translation of patients’ advance directives in the ICU, with a focus on practical difficulties and obstacles in this regard.

## Methods

A search of the literature was conducted using the online digital databases PubMed and Medline with the keywords "advance directive", "living will", "healthcare proxy", "withdrawal of care”, “withholding of care", "end-of-life decisions”, and “end-of-life care", without any restrictions on titles and/or abstracts. The initial search results were evaluated by two authors (SMB and RS) by visually screening the titles and abstracts to identify randomized trials, prospective and retrospective theme related studies published until November 2022. Further inclusion criteria were studies conducted in intensive care patients and neurocritically ill patients published in English or German. Studies on pediatric patients (< 18 years) and those published in other languages were excluded. For a study to be included, both authors had to agree on the relevance of the particular study in relation to the predefined theme of patients’ advance directive implementation in ICUs. Reference lists of identified studies were screened for additional studies complying with the inclusion criteria (citation tracking).

### Impact of advance directives in the ICU

**Fig. 1:**
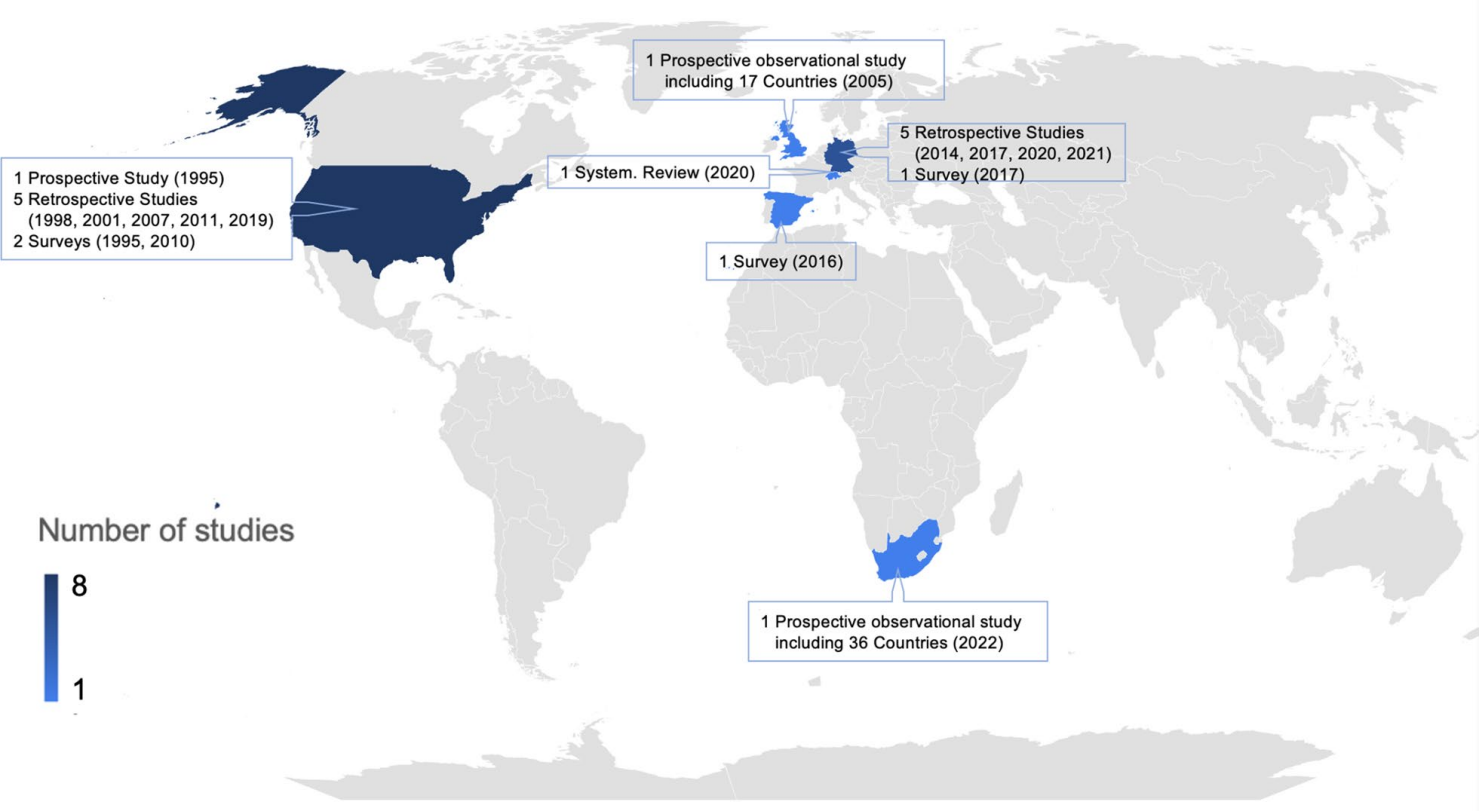
Detailed information of identified studies. Geographical overview of included studies with numbers in parentheses indicating year of publication per study

Our search yielded 17 suitable studies published between 1995 and 2022, all of which were observational and comprised 2 prospective, 3 cross-sectional, 11 retrospective studies as well as one systematic review reporting from the United States, Europe and South Africa. Table 1 presents the study characteristics, reported frequency of advance directives, and challenges that come along with the transmission and implementation of directives in the ICU. The identified studies reported investigations including 149,413 patients and 1210 health care professionals involved in surveys. Figure 1 provides detailed information on study design, publication year, and study origin. The number of studies increased over time, with 5 studies identified between 1995 and 2008 (first 14 years) and 12 studies identified between 2009 and 2022 (last 14 years). The geographical distribution is in line with a recent study that investigated end-of-life decision-making in the ICU on a global scale [7].

**Table 1:** Characteristics and key results from reviewed studies

Year; Ref	Study type	Study population	Country	Written ADs	Impact of AD	Challenges of transmission of ADs	Challenges of implementation of ADs
Prospective studies							
2022; [7]	Prospective	87,951 ICU patients, 15% died or received therapy limitation and were further analyzed	36 countries of South Africa	9% (1199 of the 15%)	N/A	N/A	N/A
1995; [19]	Prospective	26 ICU patients with ADs	USA	16%	N/A	- In 34% AD was discovered 1–12 days after ICU transfer - Controversies among care providers about meeting ADs in 34%	In 4% [1 patient] physician adhered to AD for most treatments but struggled with withdrawing MV
Cross-sectional studies							
2017; [17]	Cross-sectional study	998 ICU patients	Germany	51% (39% powers of attorney; 29% with ADs)	N/A	40% stated that they had given the relevant document to the hospital, yet such documents were present in the patient’s hospital record for only 23% (88/385 powers of attorney (23%) and 93/293 ADs (32%)	40% of ADs and 44% of powers of attorney that were present in the hospital records were poorly interpretable due to incomplete ADs
2016; [20]	Cross-sectional study	331 ICU physicians and nurses	Spain	N/A	N/A	- 91% didn't check for ADs and 90% were unaware of ADs	N/A
1995; [22]	Cross-sectional survey	879 ICU physicians	USA	N/A	N/A	N/A	- 34% of physicians would continue MV against wishes (belief in patient's chance of recovery, patient's best interest, fear of litigation or legality) - 11% of physicians refused to discontinue MV despite patient request and capability to decide
Systematic review							
2020; [15]	Systematic review	35,717 neurocritically ill ICU patients	Switzerland	39% ADs and/or healthcare agents	In reference to ADs, care was adapted in 71%, withheld or withdrawn in 58%, and resuscitation was withheld in every fourth patient	N/A	N/A
Retrospective studies							
2021; [11]	Retrospective	400 deceased neuro-ICU patients	Germany	22% (68/310) of patients who died after withdrawal or withholding of life-sustaining therapy	No difference in timing of withdrawal or withholding of life-sustaining therapy, nor in treatment intensity	N/A	N/A
2021; [16]	Retrospective	229 deceased ICU patients (123 in 2009; 106 in 2019)	Germany	9% in 2009; 26% in 2019	N/A	N/A	N/A
2020; [14]	Retrospective	489 deceased ICU or IMC patients	Germany	12%	Less likely to receive CPR and MV (by trend, therapy was more often limited and withdrawn)	N/A	N/A
2019; [13]	Retrospective	16`945 ICU patients (1536 died)	USA	40% of deceased patients	Lower odds of CPR in the last hour of life	N/A	N/A
2017; [18]	Retrospective	143 diseased stroke patients (focus on life-sustaining treatments)	Germany	29%	- DNR order implemented for one patient referring to AD - In 21/35 patients additional waiver of ICU measures was implemented, with 11 out of 21 referencing ADs - Comfort care initiated in 12/35, with 9 out of 12 referencing AD	29% had ADs, but only 25% were available	- Refusal of treatment was respected, when AD was found to be applicable, with exceptions in continuation of nutrition (1 patient) and hydration (3 patients) - 22/35 objected cardiopulmonary resuscitation, 19/35 mechanical ventilation, and 26/35 nutrition - 33/35 wanted treatment for pain or discomfort even if it hastens death - 34/35 patients had therapy limited - ADs lacked specificity, (16/35 being applicable)
2014; [12]	Retrospective	477 deceased ICU patients	Germany	13%	- No difference regarding withholding or withdrawing life-sustaining therapies, lengths of ICU stay - More DNR orders - CPR received less often	N/A	- 50% (32/64) of ADs were considered valid and factored in treatment decisions - Patients received MV, nutrition and circulatory support despite refusing it
2011; [8]	Retrospective	1121 ICU patients	USA	16%	No differences in care, end-of-life management, and outcomes	N/A	N/A
2010; [3]	Retrospective (on data of a survey)	3746 deceased patients via proxy responses (proportion treated on ICUs not reported)	USA	68% requiring decision-making and lacking the capacity (incl. power of attorney)	- Less likely to receive all care possible and more likely to receive limited treatment - Patients with a power of attorney were less likely to die in hospital	N/A	- 43% required decision-making in their final days and 70% of them lacked decision-making capacity, especially neurocritically ill - Subjects with living wills preferred limited (93%) or comfort care (96%) - The majority desiring limited (83%) or comfort care (97%) had care in line with preferences
2007; [21]	Retrospective	500 random patients (focus on life-sustaining treatments)	USA	95%	N/A	5% were mislabeled as having an AD	78% preferred to avoid general life support
2001; [10]	Retrospective	270 ICU patients	USA	27%	No differences in care, except for more DNR orders in first 72 h, shorter ICU stay/costs	N/A	N/A
1998; [9]	Retrospective	401 ICU patients	USA	5%	No differences with or without ADs	Medical personnel are unaware of AD	11% with ADs rejecting CPR received CPR

*AD* advance directive, *CPR* cardiopulmonary resuscitation, *DNR* do-not-resuscitate, *ICU* intensive care unit, *IMC* intermediate care, *MV* mechanical ventilation, *N/A* not available or reported, *US* United States

Several retrospective studies reported that advance directives had no association with the course of intensive care, as no significant differences in diagnosis, monitoring, therapy, and outcome could be observed between patients with and without advance directives [8, 9]. However, the authors of the oldest and rather small retrospective study including elderly ICU patients (≥ 65 years of age) suggested that medical staff may not have been aware of the presence of advance directives, as two patients (11%) were resuscitated despite having explicitly expressed their opposition in written form [9]. In one of the studies including 1121 oncologic ICU patients, even the presence of a healthcare proxy showed no association with the number of ICU procedures [8].

Similar findings were reported in another retrospective observational cohort study conducted at a USA cancer center’s ICU including 270 patients [10]. Among these cancer patients, patients with advance directives were found to have higher rates of do-not-resuscitate (DNR) orders within the first three days of admission, shorter ICU stays, and lower treatment costs indicating guidance by advance directives towards duration of therapy and resuscitation status. However, patients with an advance directive had no significant differences regarding the invasiveness of care, or survival rates when compared to patients without [10]. Several of these findings were corroborated by the only retrospective observational study examining a cohort of 400 neurocritically ill patients [11]. In this study the presence of advance directives or healthcare proxies was not associated with an earlier withdrawal of life-sustaining treatment, nor did it result in differences in treatment intensity.

In contrast to the aforementioned studies, several newer studies with a more valid methodology have indicated significant effects of advance directives on the extensiveness of intensive care. Three additional retrospective studies from the USA and Germany confirmed such influence of advance directives on the resuscitation status of intensive care patients [12–14]. However, the German study including 489 patients who died during their ICU or intermediate care stay, found that patients with advance directives were less likely to receive resuscitation and mechanical ventilation, with life-sustaining therapies being discontinued slightly more often than those without advance directives [14].

Our recent systematic review regarding clinical associations with the presence of advance directives in neurocritically ill patients revealed that treatment was adjusted in up to 71% of cases, with 58% of patients refusing intensive care, and one out of four patients having issued a do-not-resuscitate order [15]. Nevertheless, it is important to note that the studies included focused on neurocritically ill patients in general and not specifically on ICU patients.

Determining the underlying reasons for the inconsistent effects of advance directives on intensive care is challenging. The advance directive availability, the medical staff's awareness of the directive's existence, the directive's specific content as well as local laws and jurisdiction may all influence the quality of clinical translation.

### Transmission and prevalence of advance directives

The frequency of advance directives and challenges of their transmission as reported from the reviewed studies is presented in Table 1. The prevalence of advance directives in ICUs varies significantly across different regions [7]. A recent large-scale prospective study involving almost thirteen thousand patients from 199 ICUs across 36 countries revealed that North America has the highest prevalence of advance directives, reported to be 49.3%, followed by Central Europe (13.1%), Asia (3.9%), Australia and New Zealand (3.7%), Latin America (3%), and Northern and Southern Europe (2.6%) [7]. In contrast, advance directives were found to be nonexistent in African ICUs (0%) [7]. According to a retrospective cohort study conducted in the surgical ICUs of a German university hospital between 2009 and 2019, the prevalence of advance directives seems to be increasing over time [16].

In light of a law mandating healthcare providers to respect advance directives in all medical decision-making, irrespective of the patient's illness stage, the study revealed a substantial threefold rise in the prevalence of advance directives, increasing from 8.9% in 2009 to 26.4% in 2019 [16]. Nonetheless, the study does not definitively establish a causal link between this increase and the implementation of the new law or heightened awareness. According to a German cross-sectional study of 2017, age, presence of a life-threatening illness, experiences related to such illnesses, presence of children, and marital status have been identified as patient-related factors that influence the likelihood of having an advance directive [17].

Despite this slow yet promising progress, several challenges persist in the effective transmission of advance directives that may hinder their impact in the ICU setting.

One of the primary challenges in the implementation of advance directives in the ICU appears to be that documents are either not transmitted or lost during their medical teams’ transmission. In the German cross-sectional study, which included 998 intensive care patients at an academic teaching hospital, 512 patients (51.3%) reported having an advance directive or a health care proxy [17]. Of these patients, only 203 (39.6%) provided the corresponding document to the attending medical team, and advance directives were only available in the medical records of 93 patients (31.7%). Notably, the study excluded patients with poor outcomes, which possibly further influenced the results [17]. These concerning results are consistent with another retrospective study from Germany looking at advance directives in acute stroke patients treated in a stroke unit [18]. Out of 143 patients who died of ischemic or hemorrhagic stroke, advance directives were ultimately only available in 24.5% of patients, despite 29.4% of patients reporting the possession of an advance directive [18]. Nevertheless, the authors noticed a progressive rise in the availability of directives during the course of the observation period underlining the importance of raising awareness. The raised availability issues of advance directives are worrisome as the provision of intensive care according to a patient’s values and will is of imminent importance for humane intensive care. As shown, advance directives are important points of reference and can impact the quality and intensity of critical care, increasing the compliance of the medical treatment with the patients’ wills.

Not only failure to transmit written directives, but also delayed transmission to healthcare providers during critical care poses additional challenges and can lead to inappropriate or undesirable intensive care measures, increased utilization of intensive care resources, and unnecessary decision-making burdens for patients, family members, and medical staff. However, there is currently limited evidence supporting this assumption. Our screening of the literature identified only one prospective observational study from 1995 in this context [19]. This study focused on 26 patients with advance directives who were admitted to a tertiary cancer center's ICU. Of these patients, nine experienced delays of 1–12 days in the transmission of their advance directives. The delay was mainly caused by patients or relatives, who often transmitted the advance directives reluctantly and only when the patient’s health had deteriorated considerably [19]. A survey from Spain including 331 experienced critical care specialists highlighted that the medical team’s good clinical practice regarding advance directives is of imminent importance [20]. The survey revealed that 90.6% of respondents did not verify whether their patients had an advance directive, while 90.3% were unaware of the specific measures mentioned or specified in the directives [20]. Physicians, however, representing one-fifth of the respondents, exhibited a higher level of awareness regarding the existence of advance directives and demonstrated a greater likelihood of adherence to them in emergency situations as compared to the responding nurses. Notably, 50.2% of all respondents reported that advance directives are usually not respected, a finding in strong contrast to the fact that 82.8% indicated that such directives are useful in guiding treatment decisions [20].

Overall, the lack of evidence regarding the clinical effects of insufficient accessibility of advance directives, and the insufficient awareness among ICU doctors and nurses about the existence of such directives underscores the urgent need for further research in this area. Such research could identify potential consequences, establishing clinical guidelines and policies, and enhancing outcomes for ICU patients by guaranteeing that their preferences and values are honored in times of critical illness. The latter could best be targeted by interventional studies aiming to improve the healthcare professionals’ awareness as well as the accurate translation of patients’ directives into clinical practice. In order to design such studies, deeper knowledge of the current data regarding the implementation of advance directives during intensive care treatment is necessary, which will be discussed in the following section.

Of note, our search of the literature did not identify any studies examining the clinical effects of the implementation of a systematic screening for patients’ advance directives or a systematic screening and translation of the content of existing directives into clinical practice.

### Implementation of advance directives in the ICU

An overview of the challenges that come along with the implementation of advance directives in the ICU is compiled in Table 1. The implementation of patients’ advance directives comes with additional challenges, including issues with their applicability, phrasing, and compliance with the advance directive by the critical care team and family members.

A recent retrospective study conducted in Germany analyzed 477 patients who died in the ICU of a university hospital between 2010 and 2011 [12]. The study found that only 13% of patients had advance directives. Out of these, only 50% were considered valid and factored in treatment decisions, as in the remaining 50%, clinicians found that the clinical conditions did not fulfill the hypothetical criteria regarding the health status prespecified in the directive. Contrarily, a retrospective review of medical records showed that the health status of 89% of patients with directives met the medical conditions described in the directive [12]. This questions the clarity of prespecified medical conditions under which advance directives take effect, given that half of the existing advance directives are simply not translated into intensive care because descriptions often leave room for interpretation [12]. In the same study the vast majority of advance directives rejected life-prolonging measures, such as artificial nutrition, resuscitation, mechanical ventilation, and intensive care [12]. The only measure desired by most patients with an advance directive was adequate pain management. Even after taking the advance directive into account, life-sustaining measures differed from the actual therapy provided. Of 21 patients who rejected mechanical ventilation in their advance directives, 13 patients (62%) were still receiving mechanical ventilation at the time of death. Seven of 22 patients (32%) were artificially fed, although they objected artificial feeding in the advance directive, and eight (26%) of 31 patients were receiving circulatory support at the time of death against their prespecified will. Interestingly, the ratio of treatments received to treatments refused in patients with a questionable applicable advance directive differed little from the ratio of patients with an unequivocal advance directive [12]. Only the decision to forgo cardiopulmonary resuscitation and discontinue treatment was slightly more common among patients with an advance directive. In general, patients with an advance directive had more DNR orders and were resuscitated less frequently than patients without directives. The authors also point out that due to the retrospective nature of this study, they were unable to assess whether treatments that did not comply with advance directives were in fact treatments that patients did not want at that particular time or if patients verbally expressed otherwise [12].

Moreover, patients’ advance directives frequently lack specificity regarding specific medical conditions and treatments they should be applied to. A Mayo Clinic survey analyzing 500 randomly selected advance directives from 2004 to 2005 showed that directives often reject life-prolonging measures without providing further details [21]. This might be due to insufficient knowledge about intensive care treatments and possibilities. In addition, advance directives frequently contain subjective wording to describe the health conditions in which the advance directive should be applied. Descriptions such as "advanced impairment of brain function" or "imminent death" leave the treating team a lot of room for interpretation [12].

In contrast, not ICU restricted data involving adults over 60 years of age who had died, revealed that 83.2% of patients who requested limited care and 97.1% of patients who requested comfort care received care consistent with their preferences [3].

Insights regarding the implementation of advance directives in neurocritically ill patients come predominantly from outside the ICU sector. For example, a retrospective observational study conducted in a German stroke unit with 143 patients found that most of the 35 available advance directives (24.5%) rejected resuscitation, mechanical ventilation, and artificial nutrition [18]. Less frequently, it was stated that hydration (11.4%), antibiotics (5.7%), or hemodialysis (2.9%) were not desired, and blood transfusions were rarely mentioned. Pain relief was desired in 94.3% of patients, even if it would accelerate the dying process. When advance directives were considered applicable, they were mostly respected. An exception was the ongoing hydration in 18.8% of patients, despite their explicit refusal. Comfort therapy was provided to 34.3% of patients, nine of whom were in the context of the present directive [18].

Data regarding the adherence to living wills from the United States are similar, where they are most widely used, are similar, according to a 1990 survey of 879 members of the Critical Care Section of the American Thoracic Society [22]. The survey indicated that 294 of these physicians (34%) would continue mechanical ventilation even though the patient or family clearly disapproved. Reasons included a belief that the patient had a real chance of recovery (*n* = 227, 77%), that the family may not have decided in the patient's best interest, (*n* = 115 physicians, 39%), physician’s fear of litigation (*n* = 55 physicians, 19%), or doubts whether termination of life support was legal (*n* = 42 physicians, 14%). In fact, 33 physicians (11%) refused to discontinue mechanical ventilation even though the patient had requested it and was capable of making that decision [22].

## Conclusions

**Fig. 2:**
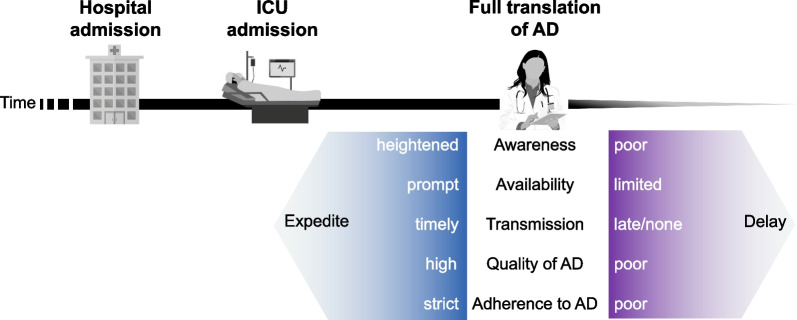
Challenges regarding the identification, transmission and implementation of patients’ advance directives during critical care. *ICU* intensive care unit, *AD* advance directive

In a time of nearly endless treatment possibilities for critically ill patients, advance directives are the only way for some patients to receive treatment according to their prespecified and autonomous will. Although the literature indicates that patients’ advance directives have the potential to improve the quality of intensive care and end-of-life treatment in the ICU, their implementation faces various challenges as compiled in Fig. 2. These challenges include insufficient knowledge, lack of awareness, inadequate or poor communication between healthcare providers and patients or their surrogates, lack of standardization of directives, and ethical and legal concerns. Due to the narrative nature of this review, it is important to note that these limitations are likely not exhaustive and that they may vary depending on institutional and individual circumstances. In general, the level of evidence is very low, with the most studies on the topic being retrospective chart reviews. Thus, further research is urgently needed to gain a more comprehensive understanding of the implications of these challenges and to identify specific interventions in order to optimize identification and timely translation of advance directives, thereby ensuring that patients receive the care they desire, even in the most challenging circumstances.

## Data Availability

Not applicable.

## Abbreviations

AD: Advance directives
CPR: Cardiopulmonary resuscitation
DNR: Do-not-resuscitate
ICU: Intensive care unit
IMC: Intermediate care
MV: Mechanical ventilation
US: United States
